# Supplementary material for: Determinants of HIV-1 reservoir size and long-term dynamics during suppressive ART
Source: Nat Commun. 2019 Jul 19;10:3193. doi: 10.1038/s41467-019-10884-9 (PMC6642170; doi:10.1038/s41467-019-10884-9)
Supplement: Supplementary file 1 — Supplementary Information [file 41467_2019_10884_MOESM1_ESM.pdf]

SUPPLEMENTARY INFORMATION: DETERMINANTS OF HIV-1 RESERVOIR SIZE AND LONG-TERM DYNAMICS DURING SUPPRESSIVE ART

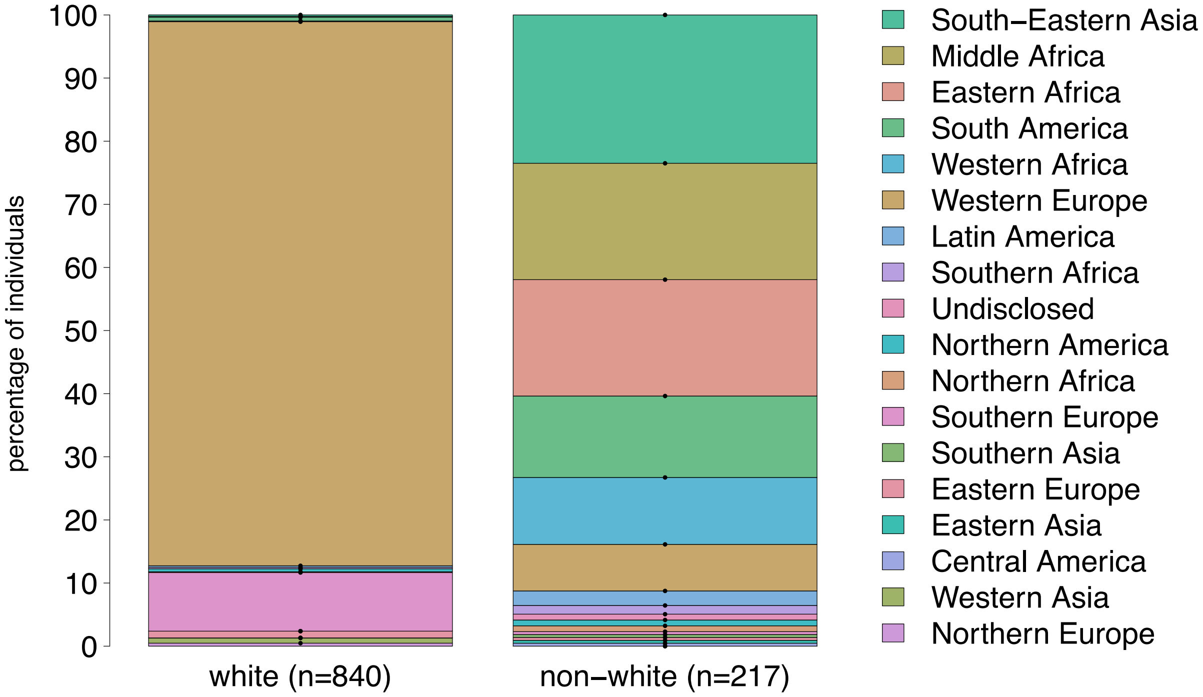

Supplementary Figure 1: Regions of origin of study participants. *724 (86.2%) individuals with white ethnicity were from Western Europe. The largest group of non-white individuals were from South-Eastern Asia (n = 51, 23.5%), followed by Middle Africa (n = 40, 18.4%), Eastern Africa (n = 40, 18.4%), South America (n = 28, 12.9%), and Western Africa (n = 23, 10.6%).*

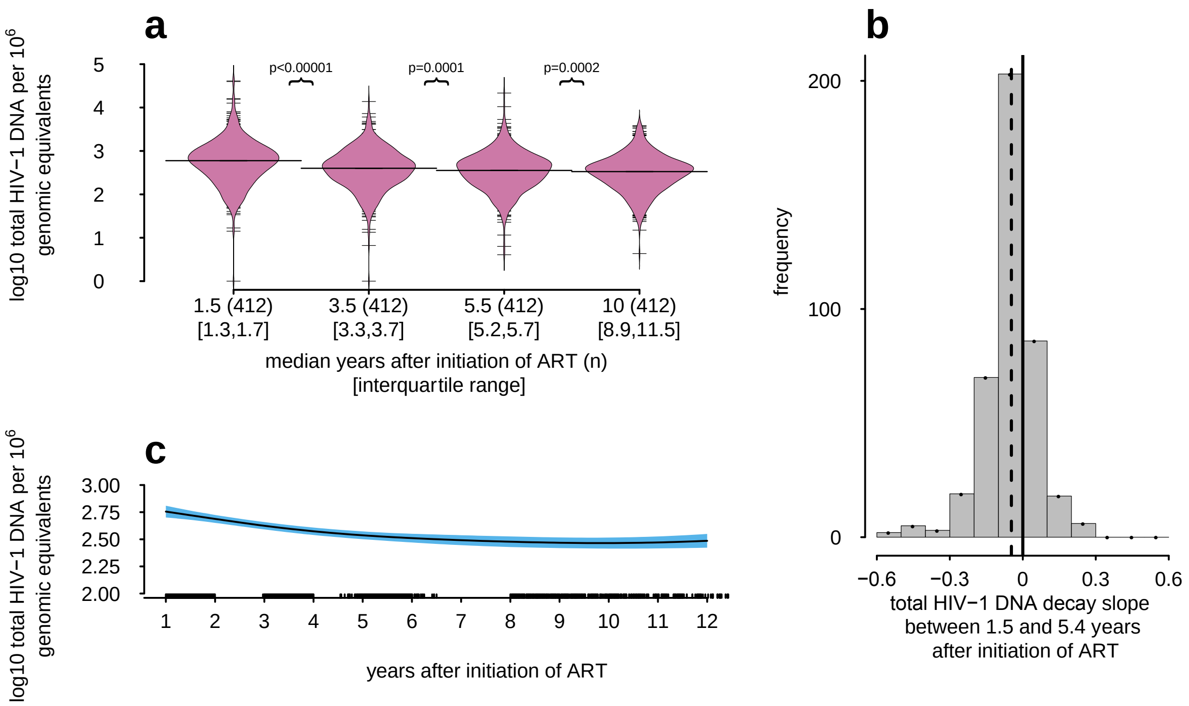

Supplementary Figure 2: The HIV-1 reservoir size and long-term dynamics in 412 individuals on suppressive ART for on median 10 years. (a) *Beanplot of total HIV-1 DNA levels in 412 individuals on long-term suppressive ART at 4 different time points (with median 1.5, 3.5, 5.5, and 10.0 years after initiation of ART) and the respective sample size. The p-values were calculated using paired Wilcoxon tests. The individual observations are shown as small lines (gray or black) in a one-dimensional scatter plot. Overlaid is the estimated density of the distributions (filled in pink) and the median is depicted by a black line.* ***(b)*** *Histogram of linear regression slope over the four measurements of total HIV-1 DNA levels with median 1.5-10 years after initiation of ART.* ***(c)*** *Spline fitted to all log10 total HIV-1 DNA/1 million genomic equivalents showing the 95% confidence intervals in blue and sampling times after initiation of ART in years on the x-axis. ART, antiretroviral therapy.*

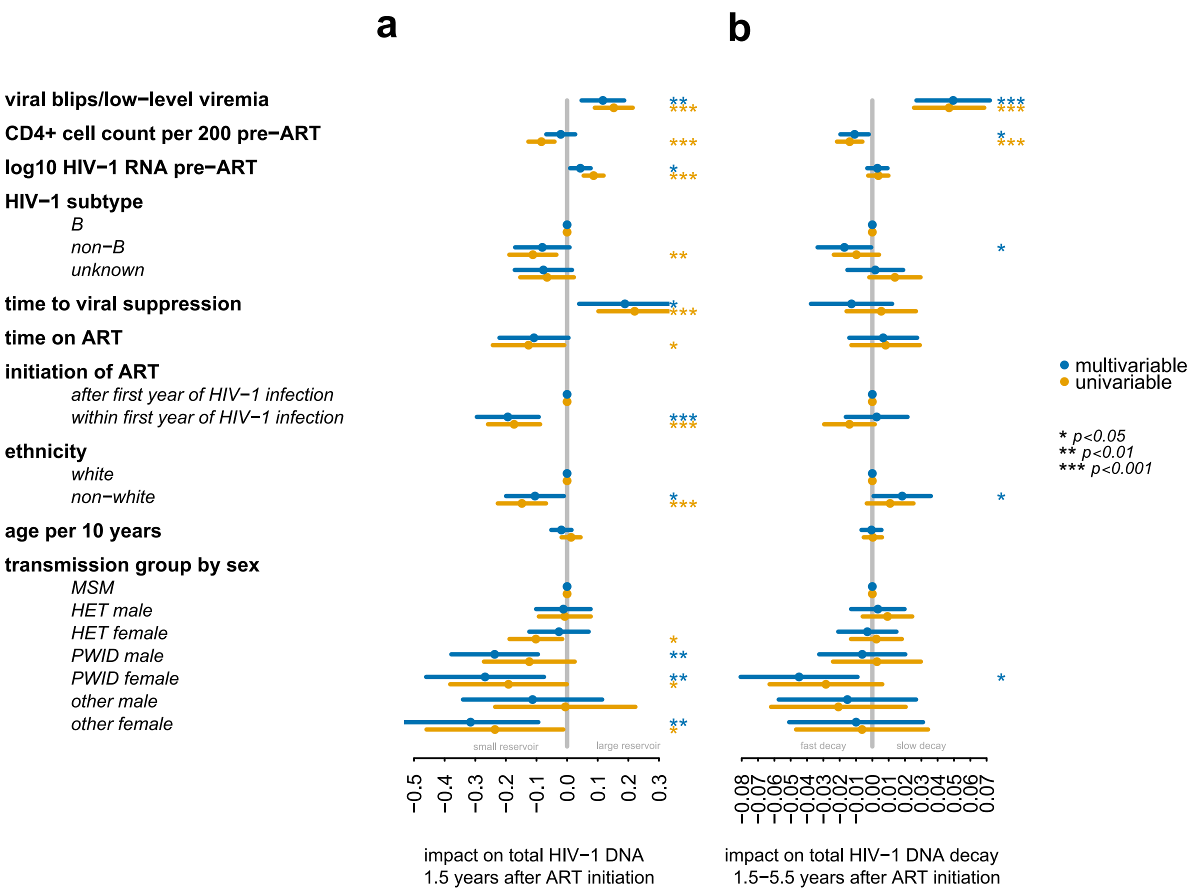

Supplementary Figure 3: Viral blips and low-level viremia numerically combined as a determinant of HIV-1 reservoir size and long-term dynamics (in the respective time divided by the number of viral load measurements)*. We defined viral load to be suppressed when all measurements were <50 HIV-1 RNA copies/ml plasma. We defined viral blips to be present when there were measurements ≥50 HIV-1 RNA copies/ml plasma, which were preceded and followed by measurements <50 HIV-1 RNA copies/ml plasma. Any subsequent viral load measurement ≥50 HIV-1 RNA copies/ml plasma within 30 days of a viral blip was considered to be part of the same viral blip. Individuals who had multiple consecutive viral load measurements ≥50 HIV-1 RNA copies/ml plasma (without experiencing virological failure as defined by two consecutive viral load measurements >200 HIV-1 RNA copies/ml plasma) were considered to exhibit low-level viremia. (****a****) Coefficient plot showing covariables associated with total HIV-1 DNA levels 1.5 years after initiation of ART and 95% confidence intervals. Viral load <50 HIV-1 RNA copies/ml plasma or low-level viremia refer to the time from 180 days after initiation of ART to the first HIV-1 DNA quantification. Reference was defined as initiation after first year of HIV-1 infection, transmission group MSM, white ethnicity and HIV-1 subtype B. (****b****) Coefficient plot showing covariables associated with the decay of total HIV-1 DNA levels and 95% confidence intervals. Corrected for initial HIV-1 DNA levels using a spline. Viral load <50 HIV-1 RNA*

copies/ml plasma or low-level viremia or blips refer to the time between the first and third sample, i.e. 1.5-5.4 years after initiation of ART. Baseline as in panel a. ART, antiretroviral therapy; MSM, men who have sex with men; HET, heterosexual; PWID, people who inject drugs; transmission group other includes unknown and transfusion; time to viral suppression refers to time taken for viral load to drop below 50 HIV-1 RNA copies/ml plasma; CD4+ cell count was measured per 200 cells/µl blood.

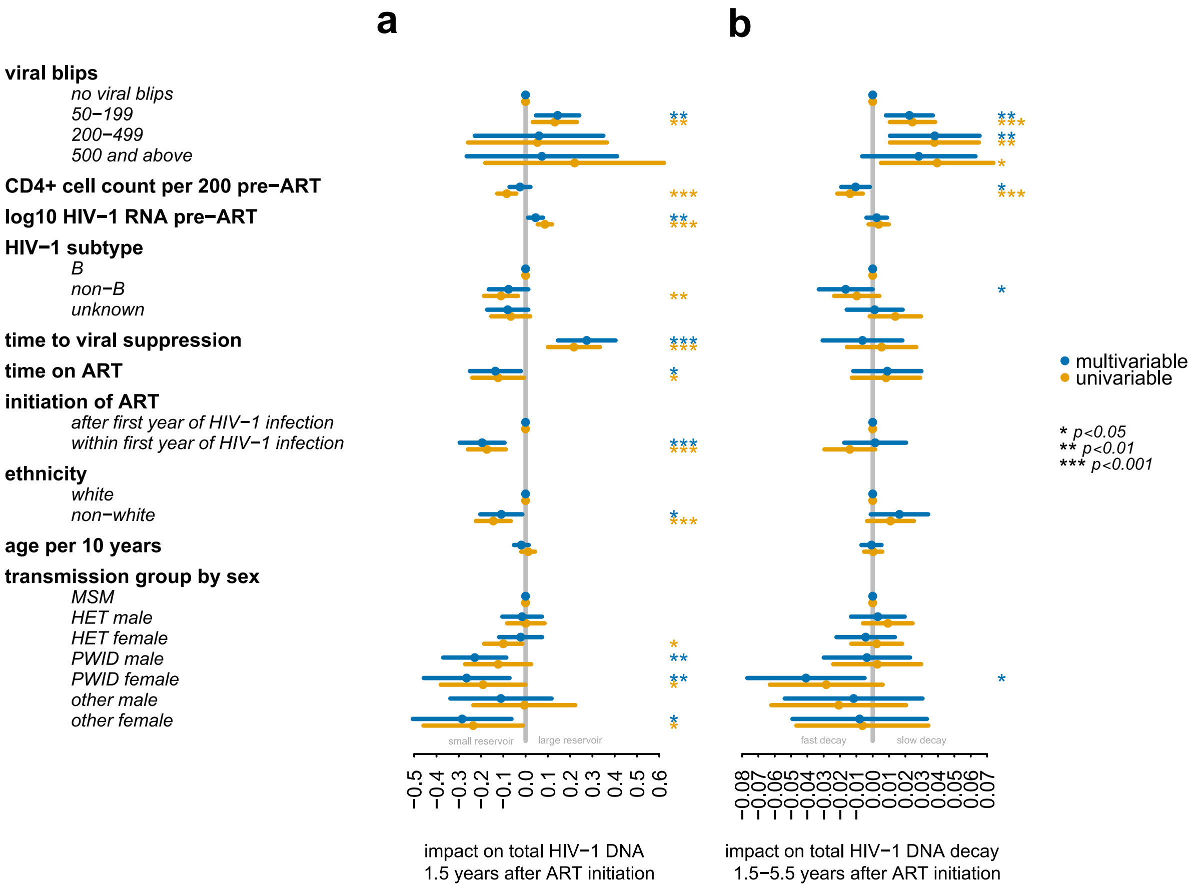

Supplementary Figure 4: Size of viral blips as a determinant of HIV-1 reservoir size and long-term dynamics. – Viral blips were stratified by the size of the largest viral blips (categories: no blips, 50-199, 200-499, 500 and above HIV-1 RNA copies/ml plasma). *(**a**) Coefficient plot showing covariables associated with total HIV-1 DNA levels 1.5 years after initiation of ART and 95% confidence intervals. Viral load <50 HIV-1 RNA copies/ml plasma or low-level viremia refer to the time from 180 days after initiation of ART to the first HIV-1 DNA quantification. Reference was defined as no viral blips, initiation after first year of HIV-1 infection, transmission group MSM, white ethnicity and HIV-1 subtype B. (**b**) Coefficient plot showing covariables associated with the decay of total HIV-1 DNA levels. Corrected for initial HIV-1 DNA levels using a spline and 95% confidence intervals. Viral load*

<50 HIV-1 RNA copies/ml plasma or low-level viremia or blips refer to the time between the first and third sample, i.e. 1.5-5.4 years after initiation of ART. Baseline as in panel a. ART, antiretroviral therapy; MSM, men who have sex with men; HET, heterosexual; PWID, people who inject drugs; transmission group other includes unknown and transfusion; time to viral suppression refers to time taken for viral load to drop below 50 HIV-1 RNA copies/ml plasma; CD4+ cell count was measured per 200 cells/µl blood.*

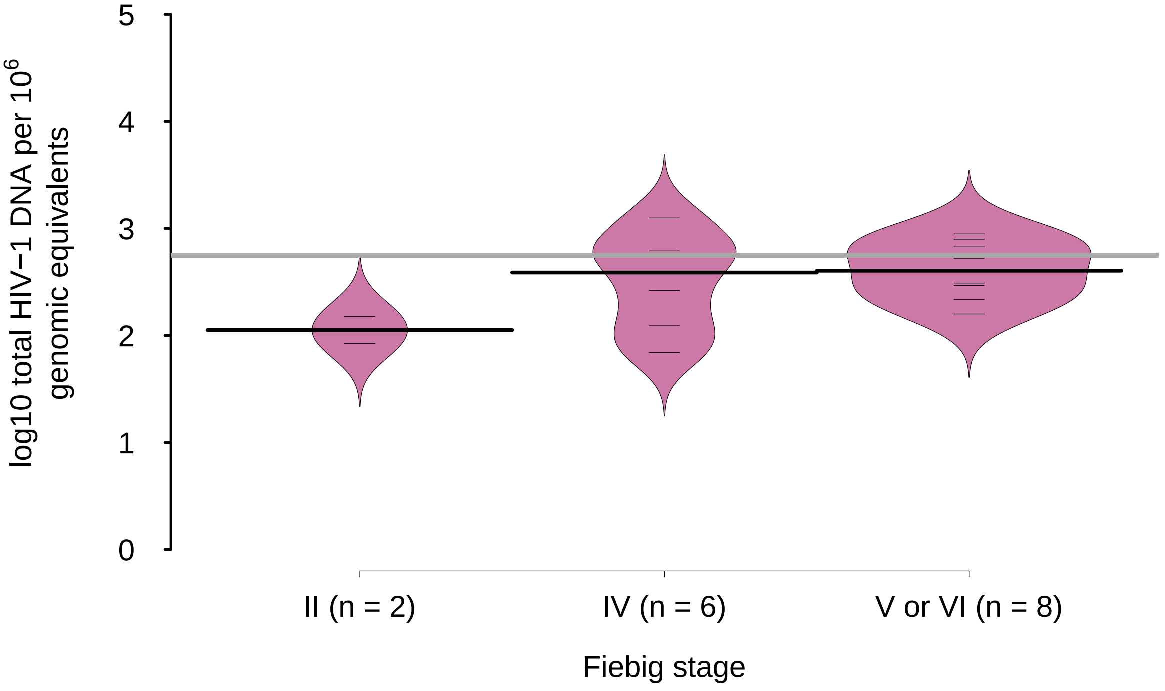

***Supplementary Figure 5: Beanplot of total HIV-1 DNA levels 1.5 years after initiation of ART stratified by Fiebig stage.** Classification of Fiebig stage was possible in 17/173 individuals starting ART within the first year of HIV-1 infection. The individual observations are shown as small black lines in a one-dimensional scatter plot. Overlaid is the estimated density of the distributions (filled in pink) and the median is depicted by a horizontal black line. The gray line indicates the population level median total HIV-1 DNA 1.5 years after initiation of ART.*

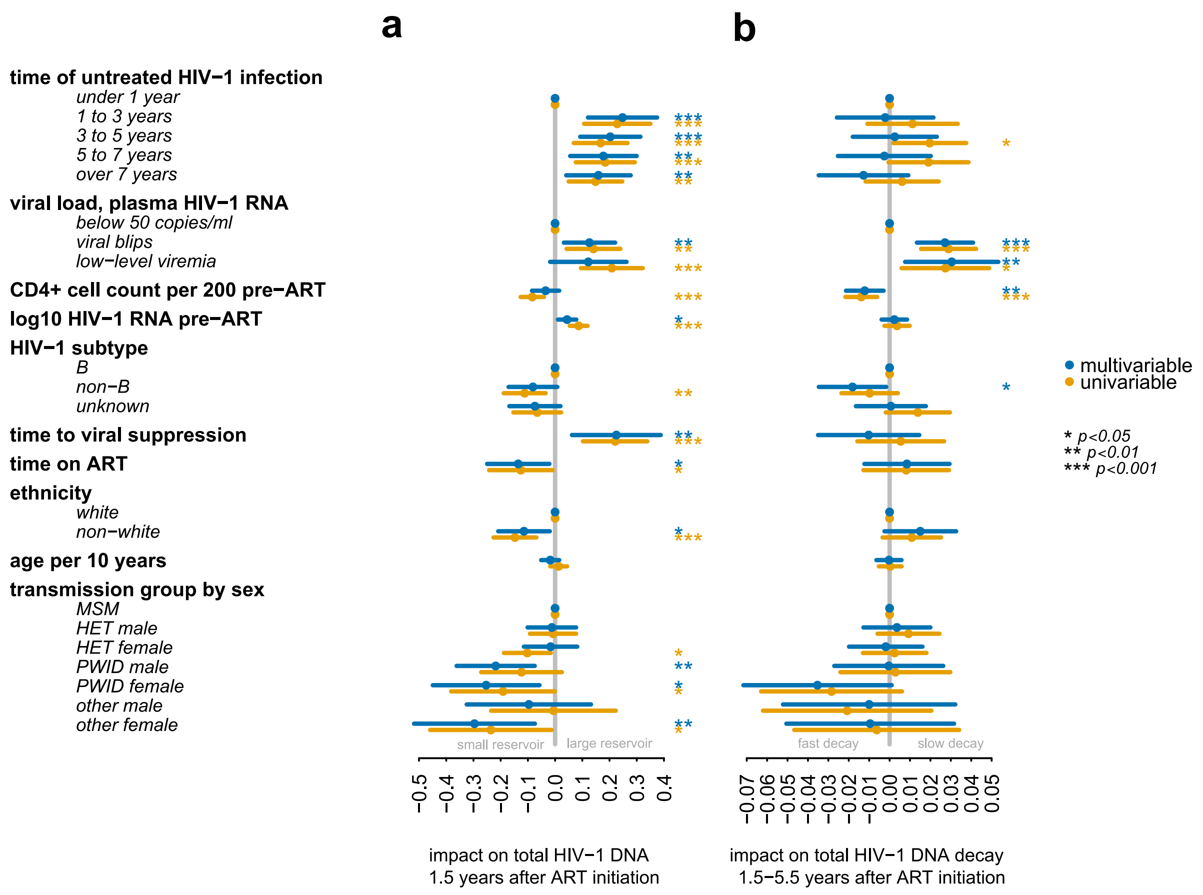

Supplementary Figure 6: Time of untreated HIV-1 infection as a determinant of HIV-1 reservoir size and long-term dynamics. Categories of time of untreated HIV-1 infection was chosen as follows: <1 year, 1 to 3 years, 3 to 5 years, 5 to 7 years, >7 years). *(**a**) Coefficient plot showing covariables associated with total HIV-1 DNA levels 1.5 years after initiation of ART and 95% confidence intervals. Viral load <50 HIV-1 RNA copies/ml plasma or low-level viremia refer to the time from 180 days after initiation of ART to the first HIV-1 DNA quantification. Reference was defined as viral load, plasma HIV-1 RNA below 50 copies/ml, time of untreated infection under 1 year, transmission group MSM, white ethnicity and HIV-1 subtype B. (**b**) Coefficient plot showing covariables associated with the decay of total HIV-1 DNA levels and 95% confidence intervals. Corrected for initial HIV-1 DNA levels using a spline. Viral load <50 HIV-1 RNA copies/ml plasma or low-level viremia or blips refer to the time between the first and third sample, i.e. 1.5-5.4 years after initiation of ART. Baseline as in panel a. ART, antiretroviral therapy; MSM, men who have sex with men; HET, heterosexual; PWID, people who inject drugs; transmission group other includes unknown and transfusion; time to viral suppression refers to time taken for viral load to drop below 50 HIV-1 RNA copies/ml plasma; CD4+ cell count was measured per 200 cells/µl blood.*

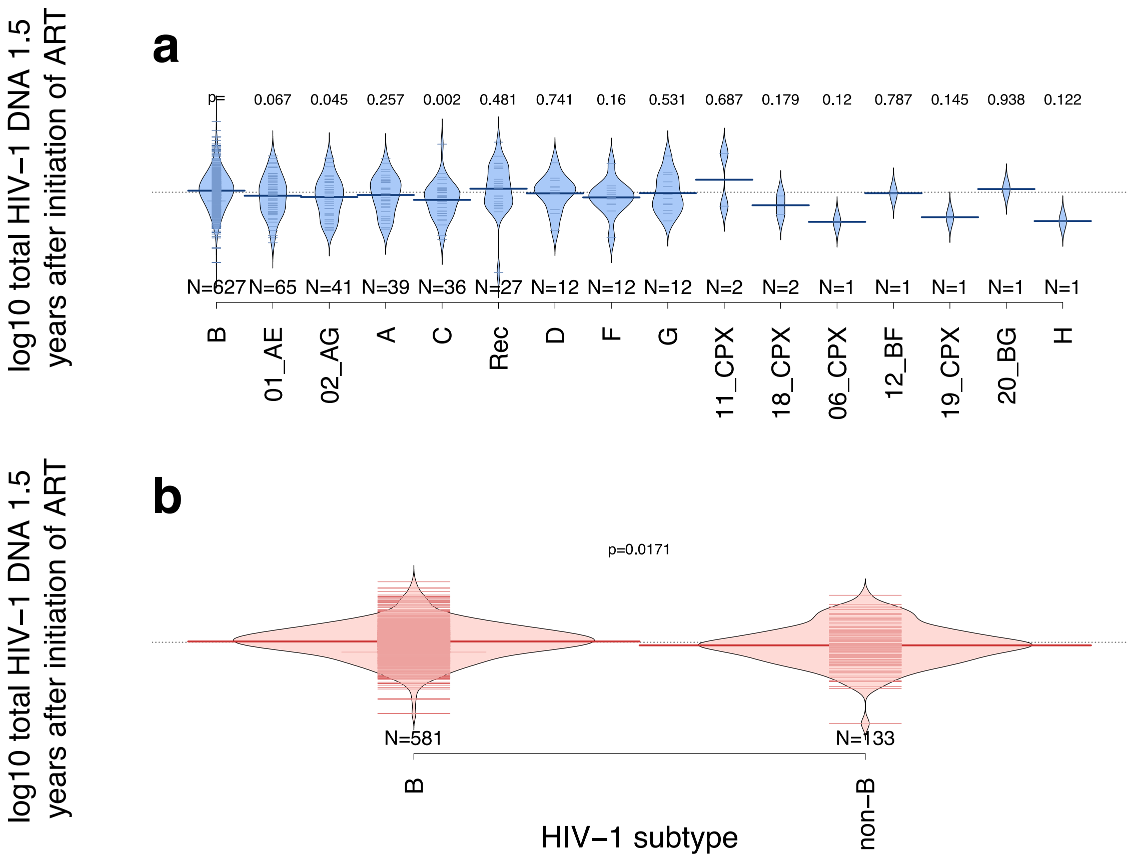

***Supplementary Figure 7: Beanplots illustrating the HIV-1 reservoir size 1.5 years after initiation of ART stratified by HIV-1 subtype. (a)** All HIV-1 subtypes, "Rec" refers to recombinant forms. **(b).** All individuals of white ethnicity stratified by infection with HIV-1 subtype B or non-B. **(a/b)** Gray continuous lines represent mean of log10 total HIV-1 DNA per 10^6 genomic equivalents in individuals infected with HIV-1 subtype B. The p-values are in comparison to HIV-1 subtype B and were derived using Wilcoxon rank sum test with continuity correction. ART, antiretroviral therapy.*

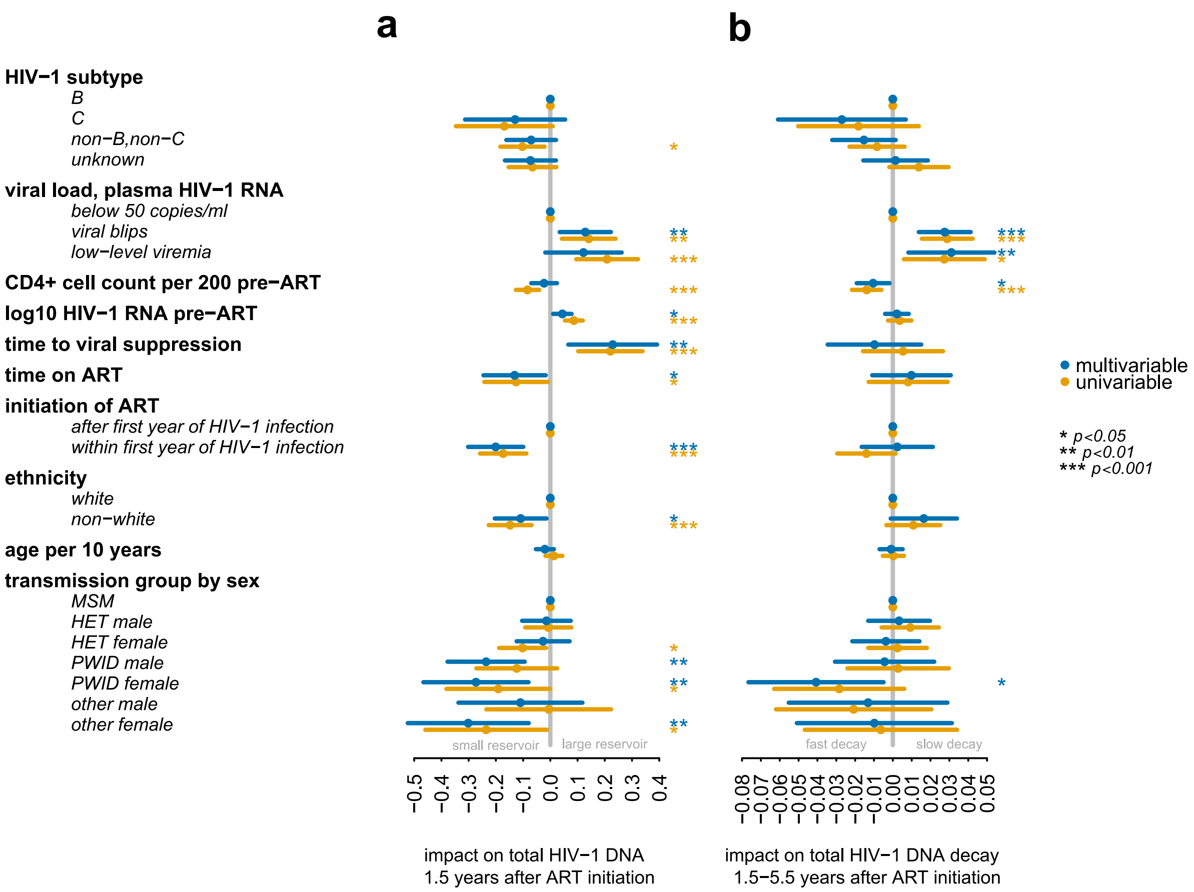

Supplementary Figure 8: HIV-1 subtype C as a determinant of HIV-1 reservoir size and long-term dynamics. (a) *Coefficient plot showing covariables associated with total HIV-1 DNA levels 1.5 years after initiation of ART and 95% confidence intervals. Viral load <50 HIV-1 RNA copies/ml plasma or low-level viremia refer to the time from 180 days after initiation of ART to the first HIV-1 DNA quantification. Reference was defined as viral load, plasma HIV-1 RNA below 50 copies/ml, initiation after first year of HIV-1 infection, transmission group MSM, white ethnicity and HIV-1 subtype B. (**b)** Coefficient plot showing covariables associated with the decay of total HIV-1 DNA levels and 95% confidence intervals. Corrected for initial HIV-1 DNA levels using a spline. Viral load <50 HIV-1 RNA copies/ml plasma or low-level viremia or blips refer to the time between the first and third sample, i.e. 1.5-5.4 years after initiation of ART. Baseline as in panel a. ART, antiretroviral therapy; MSM, men who have sex with men; HET, heterosexual; PWID, people who inject drugs; transmission group other includes unknown and transfusion; time to viral suppression refers to time taken for viral load to drop below 50 HIV-1 RNA copies/ml plasma; CD4+ cell count was measured per 200 cells/µl blood.*

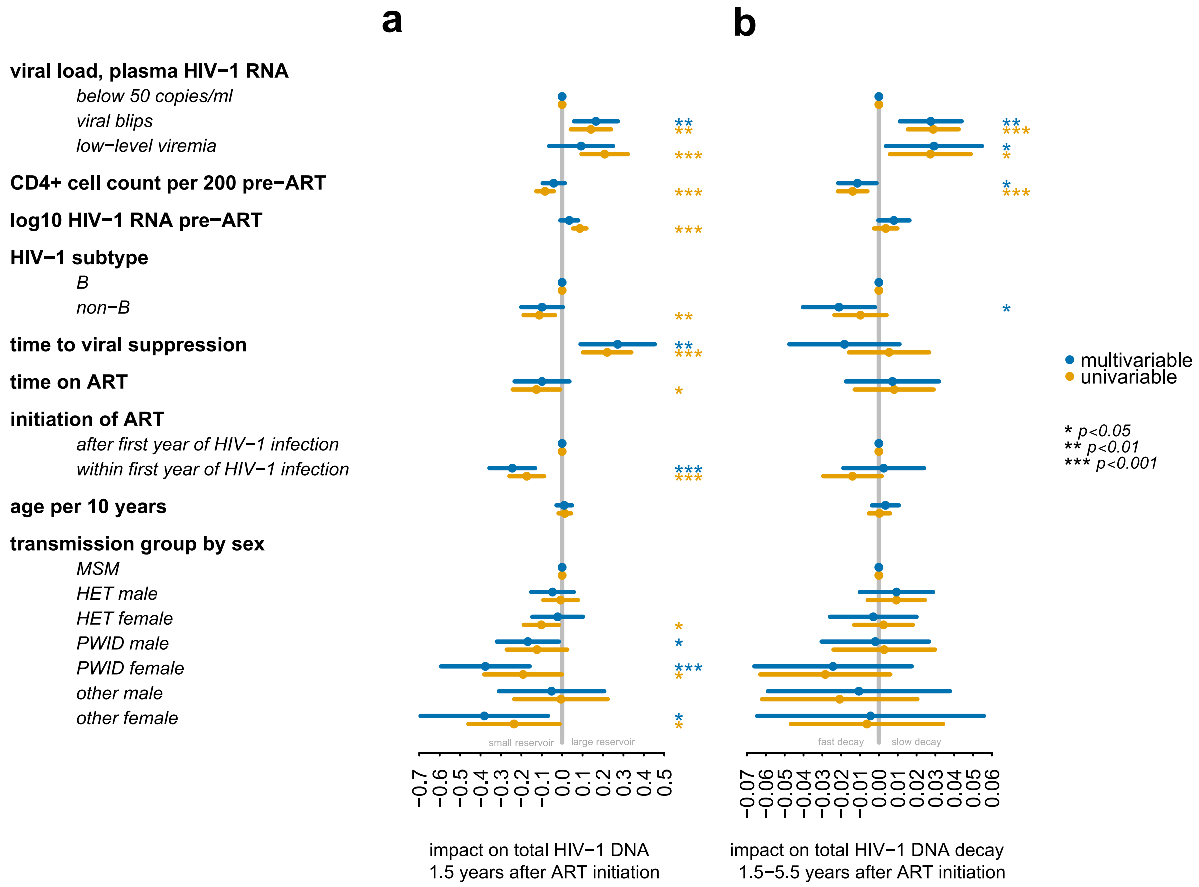

Supplementary Figure 9: Analysis of determinants of HIV-1 reservoir size and long-term dynamics as shown in Figure 3 restricted to individuals of white ethnicity and with available HIV-1 subtype information. *581 individuals were infected with HIV-1 subtype B and 133 individuals with HIV-1 non-B subtypes.* ***(a)*** *Coefficient plot showing covariables associated with total HIV-1 DNA levels 1.5 years after initiation of ART and 95% confidence intervals. Viral load <50 HIV-1 RNA copies/ml plasma or low-level viremia refer to the time from 180 days after initiation of ART to the first HIV-1 DNA quantification. Reference was defined as initiation after first year of HIV-1 infection, transmission group MSM, white ethnicity and HIV-1 subtype B.* ***(b)*** *Coefficient plot showing covariables associated with the decay of total HIV-1 DNA levels and 95% confidence intervals. Corrected for initial HIV-1 DNA levels using a spline. Viral load <50 HIV-1 RNA copies/ml plasma or low-level viremia or blips refer to the time between the first and third sample, i.e. 1.5-5.4 years after initiation of ART. Baseline as in panel a. ART, antiretroviral therapy; MSM, men who have sex with men; HET, heterosexual; PWID, people who inject drugs; transmission group other includes unknown and transfusion; time to viral suppression refers to time taken for viral load to drop below 50 HIV-1 RNA copies/ml plasma; CD4+ cell count was measured per 200 cells/µl blood.*

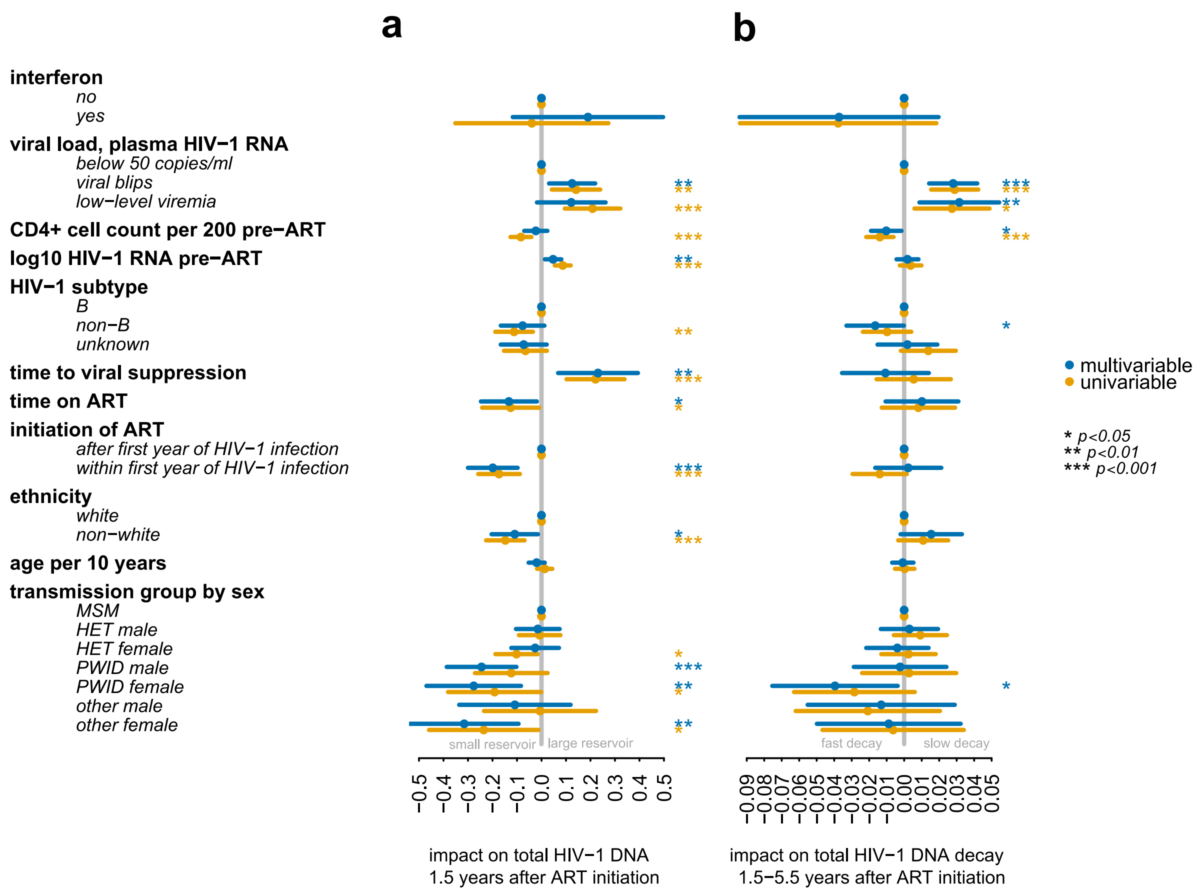

Supplementary Figure 10: Interferon treatment for HCV infection prior to the first total HIV-1 DNA sample as a determinant of HIV-1 reservoir size and long-term dynamics. *(**a**) Coefficient plot showing covariables associated with total HIV-1 DNA levels 1.5 years after initiation of ART and 95% confidence intervals. Viral load <50 HIV-1 RNA copies/ml plasma or low-level viremia refer to the time from 180 days after initiation of ART to the first HIV-1 DNA quantification. Reference was defined as no interferon treatment, viral load, plasma HIV-1 RNA below 50 copies/ml, initiation after first year of HIV-1 infection, transmission group MSM, white ethnicity and HIV-1 subtype B. (**b**) Coefficient plot showing covariables associated with the decay of total HIV-1 DNA levels and 95% confidence intervals. Corrected for initial HIV-1 DNA levels using a spline. Viral load <50 HIV-1 RNA copies/ml plasma or low-level viremia or blips refer to the time between the first and third sample, i.e. 1.5-5.4 years after initiation of ART. Baseline as in panel a. ART, antiretroviral therapy; MSM, men who have sex with men; HET, heterosexual; PWID, people who inject drugs; transmission group other includes unknown and transfusion; time to viral suppression refers to time taken for viral load to drop below 50 HIV-1 RNA copies/ml plasma; CD4+ cell count was measured per 200 cells/µl blood.*

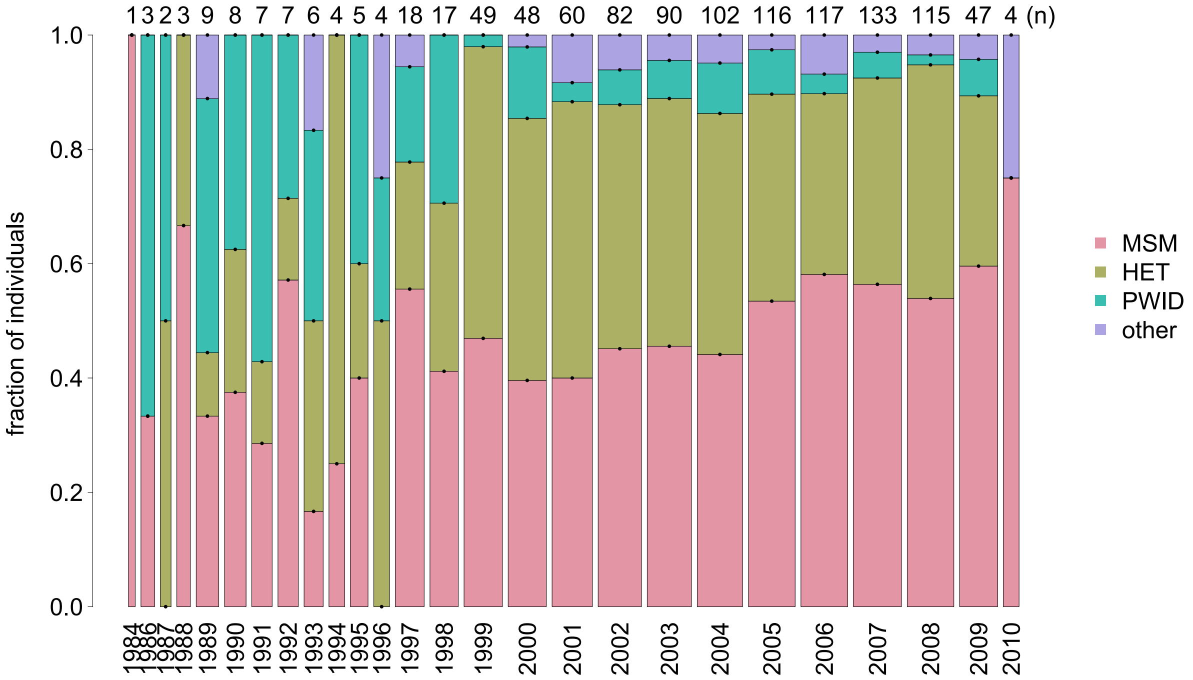

Supplementary Figure 11: Barplot showing the transmission groups per year of SHCS registration. *Width of the bars correspond to the number of registrations per year on a log scale. MSM, men who have sex with men; HET, heterosexual; PWID, people who inject drugs; transmission group other includes unknown and transfusion.*

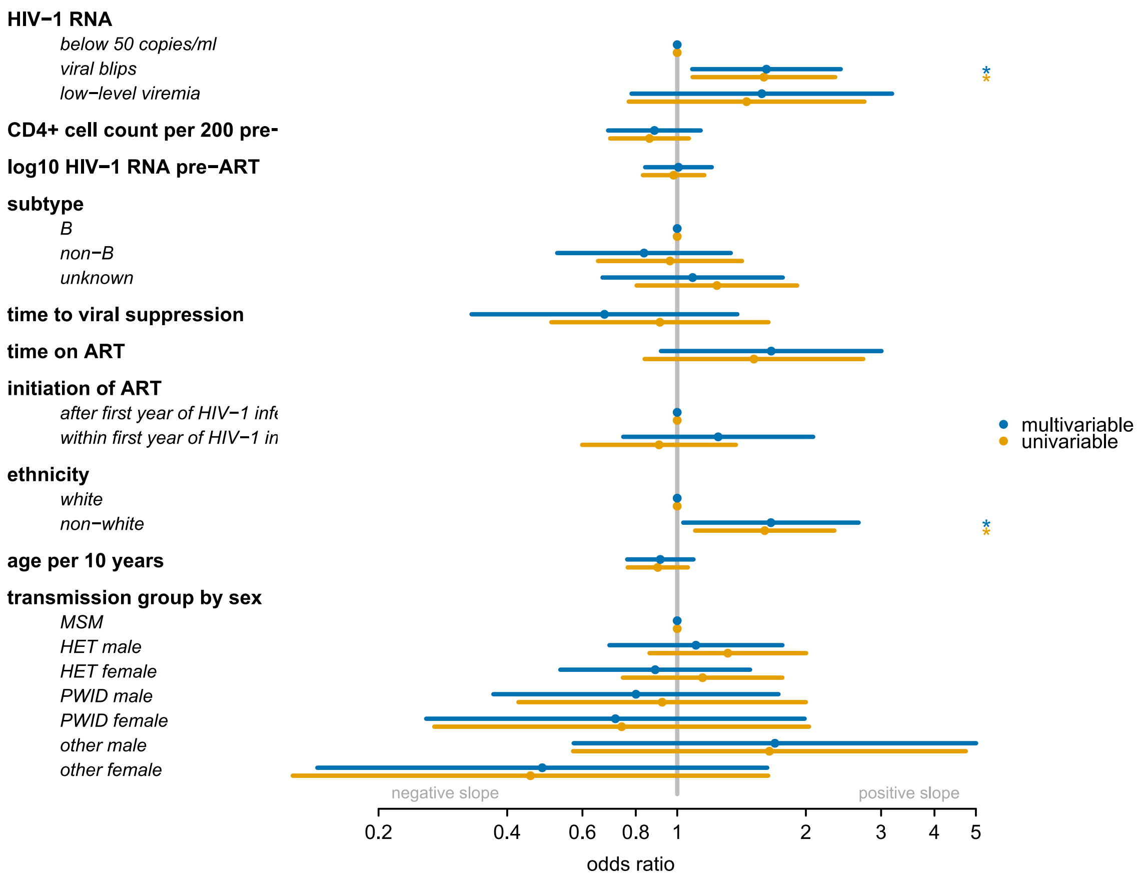

Supplementary Figure 12. Odds ratio for positive HIV-1 reservoir decay slope and 95% confidence intervals. *Corrected for initial HIV-1 DNA levels using a spline. Viral load <50 HIV-1 RNA copies/ml plasma or low-level viremia or blips refer to the time between the first and third sample, i.e. 1.5-5.4 years after initiation of ART. Baseline as in panel a. ART, antiretroviral therapy; MSM, men who have sex with men; HET, heterosexual; PWID, people who inject drugs; transmission group other includes unknown and transfusion; time to viral suppression refers to time taken for viral load to drop below 50 HIV-1 RNA copies/ml plasma; CD4+ cell count was measured per 200 cells/µl blood.*

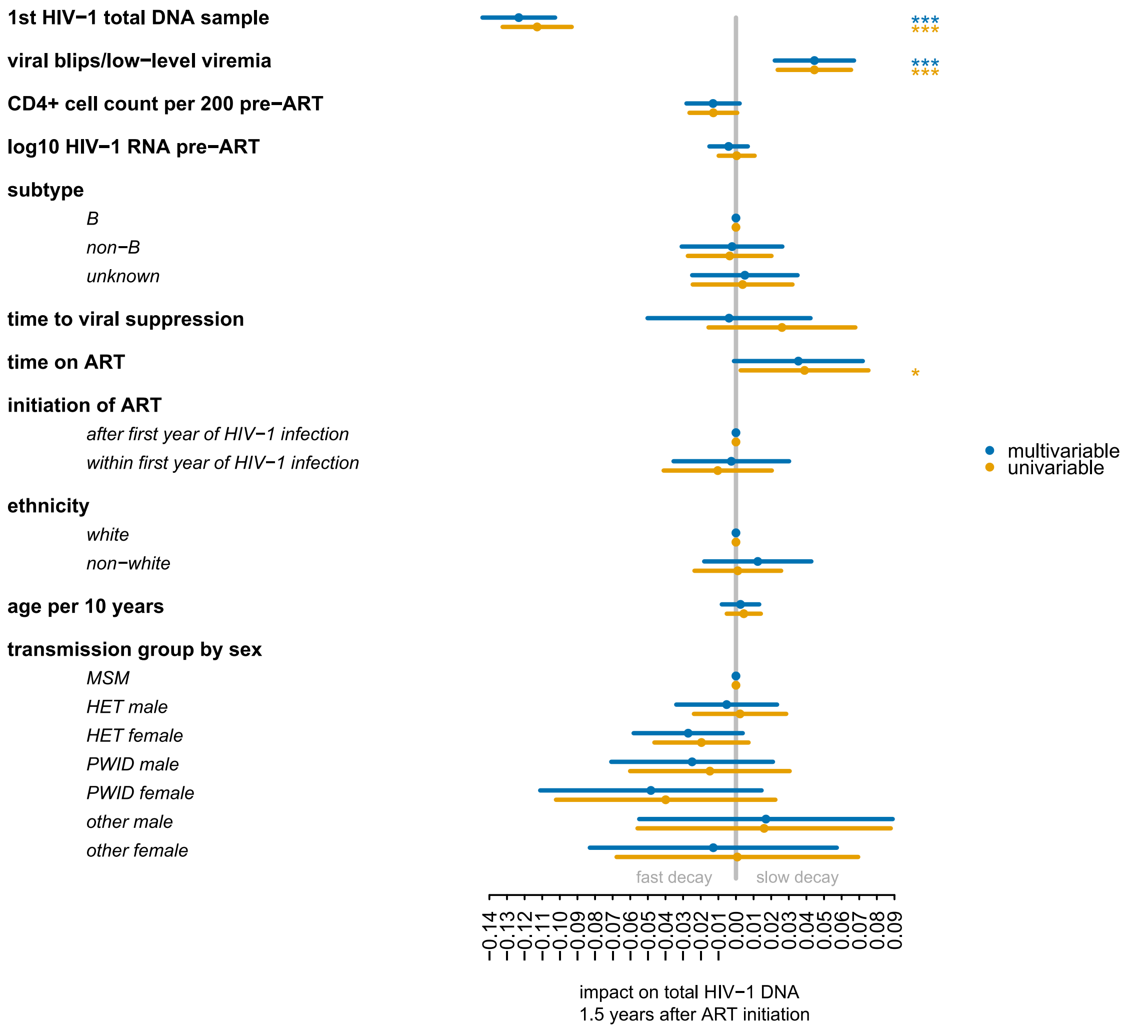

Supplementary Figure 13: Determinants of HIV-1 reservoir long-term dynamics analyzed with linear mixed effect model (implemented in the R package "lme4") and differences of total HIV-1 DNA levels compared to the first total HIV-1 DNA measurement as outcome (i.e., zero for the 1st time point, difference of 1st and 2nd measurement for the second time point, and difference of 1st and 3rd measurement for the third time point) and 95% confidence intervals. *Viral blips/ low-level viremia were numerically combined and incorporated in a time dependent manner, i.e., viral blips/low-level viremia occurring in the associated previous time period. Viral load <50 HIV-1 RNA copies/ml plasma or low-level viremia or blips refer to the time between the first and third sample, i.e. 1.5-5.4 years after initiation of ART. Reference was defined as initiation after first year of HIV-1 infection, transmission group MSM, white ethnicity and HIV-1 subtype B. ART, antiretroviral therapy; MSM, men who have sex with men; HET, heterosexual; PWID, people who inject drugs; transmission group other includes unknown and transfusion; time to*

viral suppression refers to time taken for viral load to drop below 50 HIV-1 RNA copies/ml plasma; CD4+ cell count was measured per 200 cells/µl blood.

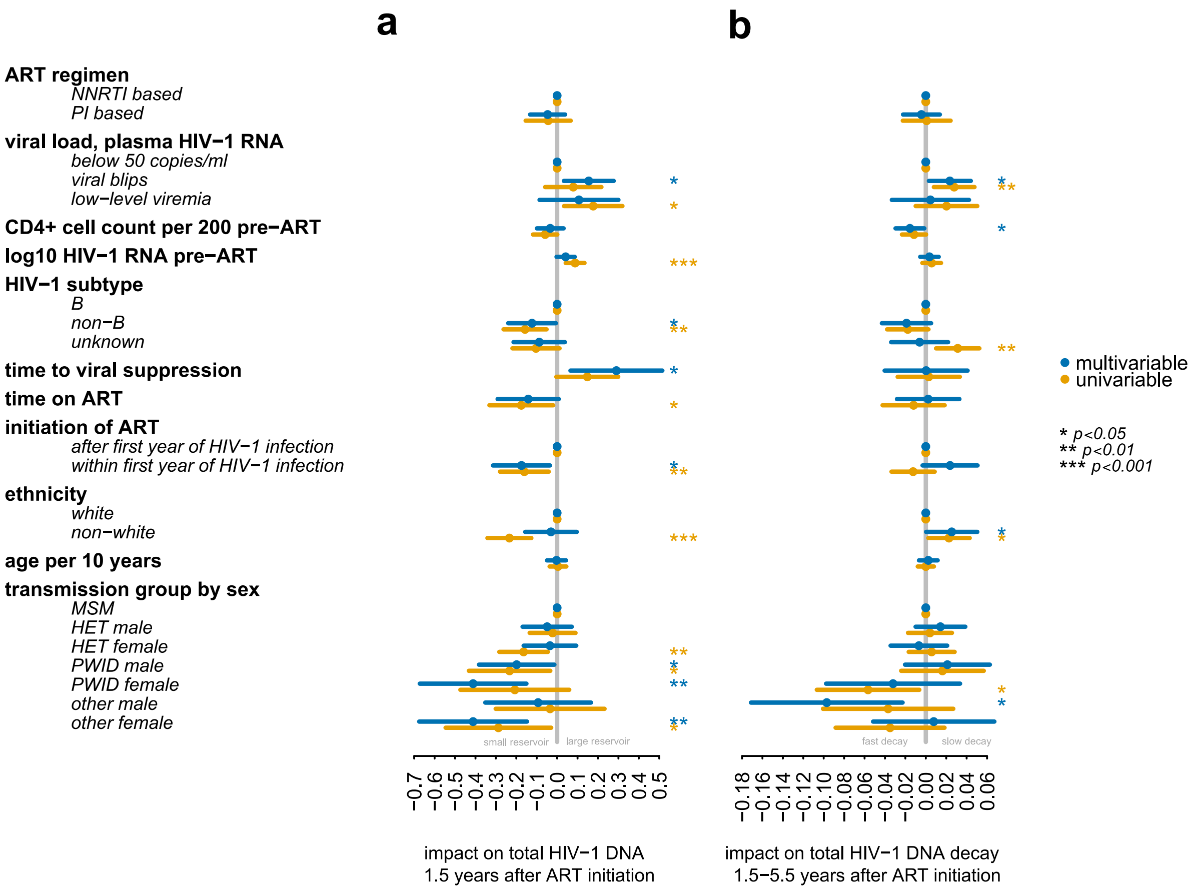

Supplementary Figure 14: The ART regimen (NNRTI based vs PI based) as a determinant of HIV-1 reservoir size and long-term dynamics. *We only considered individuals who received either a PI or an NNRTI for at least 75% of the time investigated, i.e., first 1.5 years after initiation of ART in regard to HIV-1 reservoir size and the years 1.5 – 5.4 of after initiation of ART in regard to HIV-1 reservoir long-term dynamics. Thus, we could include 659 individuals (NNRTI: n = 382, PI/r: n = 277) and 550 individuals (NNRTI: n = 358, PI/r: n = 192) in the analysis, respectively. time to viral suppression refers to time to viral load below 50 HIV-1 RNA copies/ml plasma; CD4+ cell count was measured per 200 cells/µl blood.* ***(a)*** *Coefficient plot showing covariables associated with total HIV-1 DNA levels 1.5 years after initiation of ART and 95% confidence intervals. Viral load <50 HIV-1 RNA copies/ml plasma or low-level viremia refer to the time from 180 days after initiation of ART to the first HIV-1 DNA quantification. Reference was defined as NNRTI-based regimen, viral load, plasma HIV-1 RNA below 50 copies/ml, initiation after first year of HIV-1 infection,*

*transmission group MSM, white ethnicity and HIV-1 subtype B. (**b)** Coefficient plot showing covariables associated with the decay of total HIV-1 DNA levels and 95% confidence intervals. Corrected for initial HIV-1 DNA levels using a spline. Viral load <50 HIV-1 RNA copies/ml plasma or low-level viremia or blips refer to the time between the first and third sample, i.e. 1.5-5.4 years after initiation of ART. Baseline as in panel a. ART, antiretroviral therapy; MSM, men who have sex with men; HET, heterosexual; PWID, people who inject drugs; transmission group other includes unknown and transfusion; time to viral suppression refers to time taken for viral load to drop below 50 HIV-1 RNA copies/ml plasma; CD4+ cell count was measured per 200 cells/µl blood; PI, boosted protease inhibitor.*

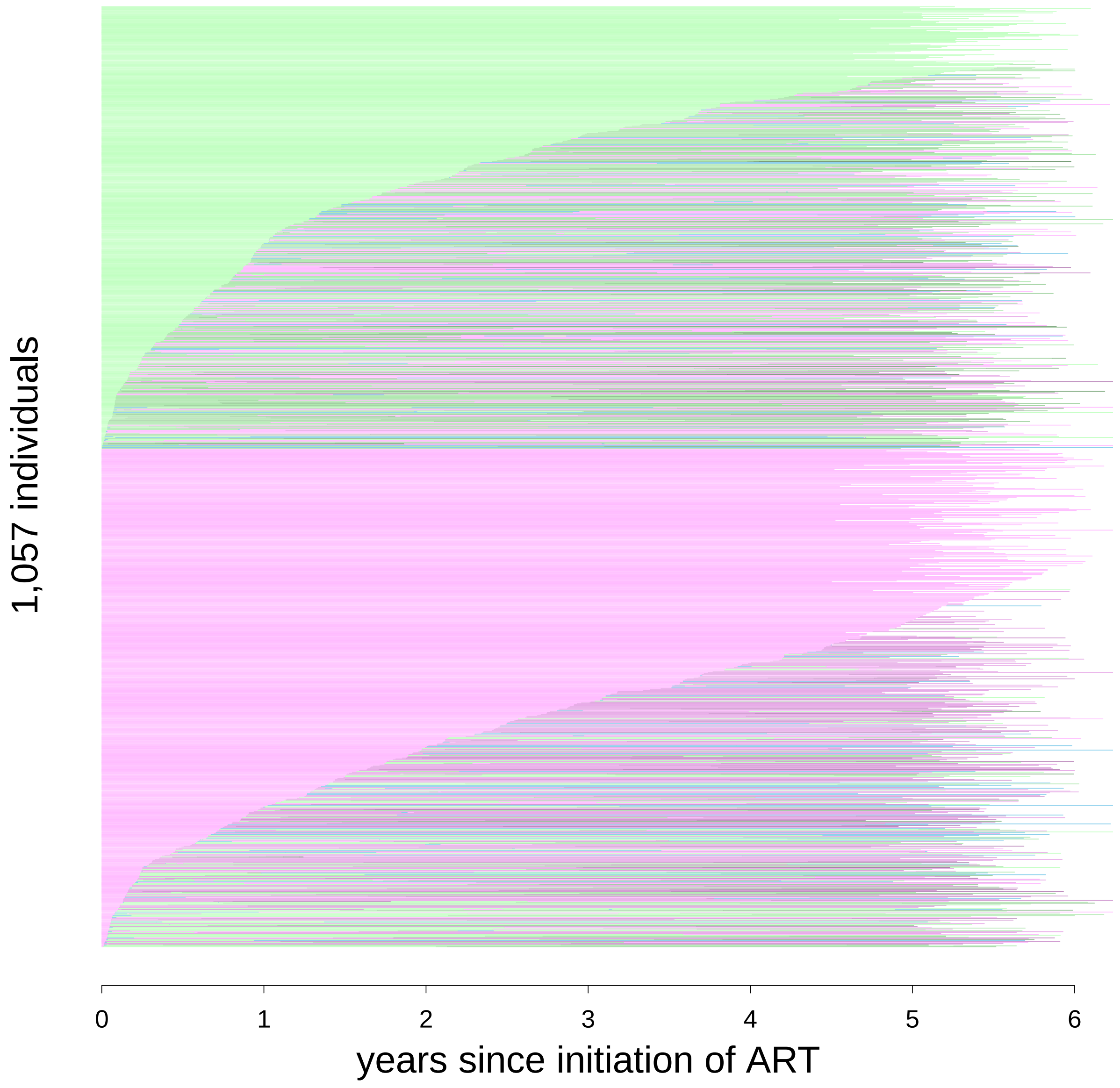

Supplementary Figure 15: Antiretroviral therapy (ART) changes within the population of 1,057 individuals. *The y-axis represents 1,057 individuals, the x-axis the time since initiation of ART. Green color represents treatment with a boosted protease inhibitor (PI) based ART regimen, purple represents treatment with a non-nucleoside reverse-transcriptase inhibitors (NNRTI) based ART regimen and blue represent treatment with an alternative regimen (mostly integrase inhibitor, few with only NRTI). Treatment switches within the same treatment class (i.e. NNRTI to NNRTI) are indicated by darkening the respective color (i.e. purple to darker purple). The individuals were ordered by the duration they stayed on the first regimen and by their first treatment regimen class.*

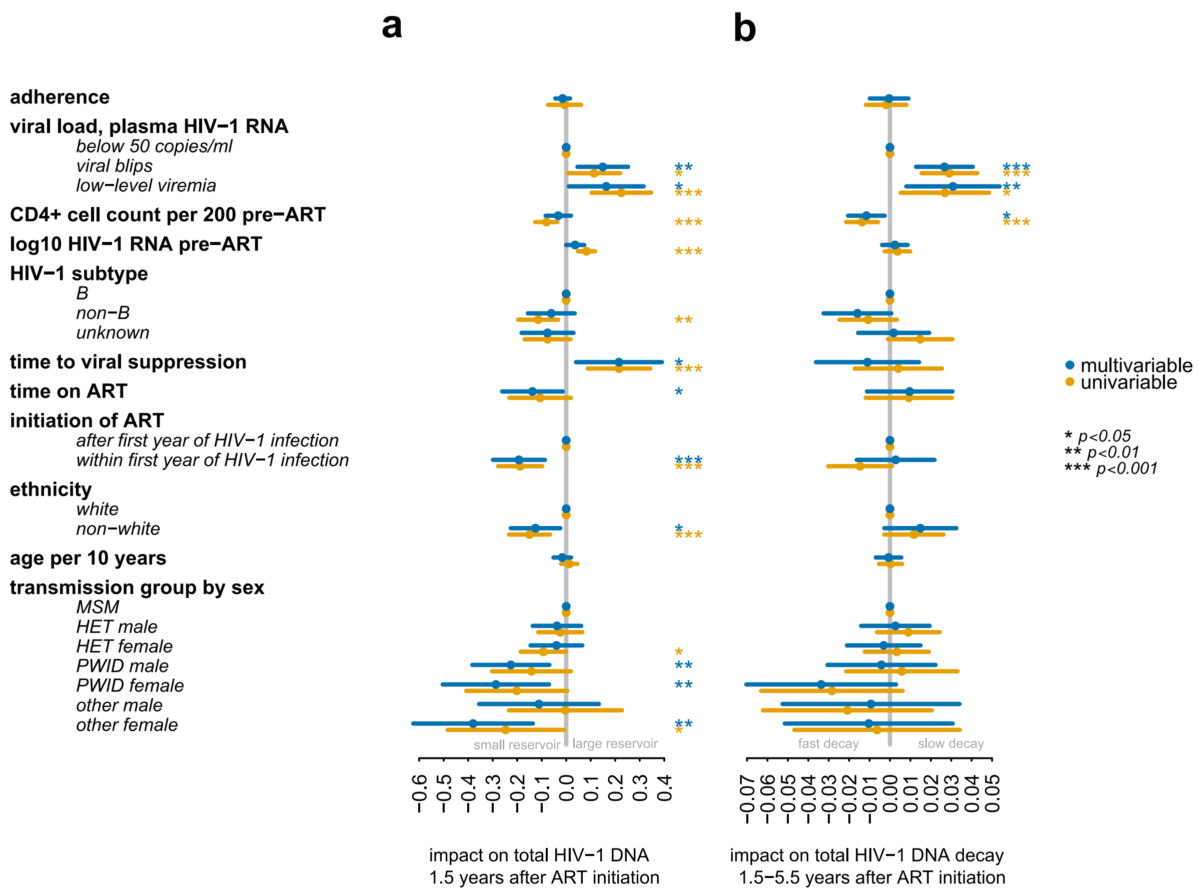

Supplementary Figure 16: Determinants of total HIV-1 DNA levels including self-reported adherence*.* *An adherence score for each patient before our first sample was taken as well as between the first and third sample. At each SHCS follow-up visit patients are asked how often a dose of ART has been missed in the 4 weeks before the visit. If they answered "every day" 28 was added to their score, if they answered "more than 1/week" 8 was added to their scores, if they answered "once a week" 4 was added to their score, if they answered "once every two weeks" 2 was added to their score, and finally if they answered "once a month" 1 was added to their score. If they reported to not have missed any ART dose 0 was added to their score.* ***(a)*** *Coefficient plot showing covariables associated with total HIV-1 DNA levels 1.5 years after initiation of ART and 95% confidence intervals. Viral load <50 HIV-1 RNA copies/ml plasma or low-level viremia refer to the time from 180 days after initiation of ART to the first HIV-1 DNA quantification. Reference was defined as viral load, plasma HIV-1 RNA below 50 copies/ml, initiation after first year of HIV-1 infection, transmission group MSM, white ethnicity and HIV-1 subtype B.* ***(b)*** *Coefficient plot showing covariables associated with the decay of total HIV-1 DNA levels and 95% confidence intervals. Corrected for initial HIV-1 DNA levels using a spline. Viral load <50 HIV-1 RNA copies/ml plasma or low-level viremia or blips refer to the time between the first and third sample, i.e. 1.5-5.4 years after initiation of ART. Baseline as in panel a. ART,*

antiretroviral therapy; MSM, men who have sex with men; HET, heterosexual; PWID, people who inject drugs; transmission group other includes unknown and transfusion; time to viral suppression refers to time taken for viral load to drop below 50 HIV-1 RNA copies/ml plasma; CD4+ cell count was measured per 200 cells/µl blood.

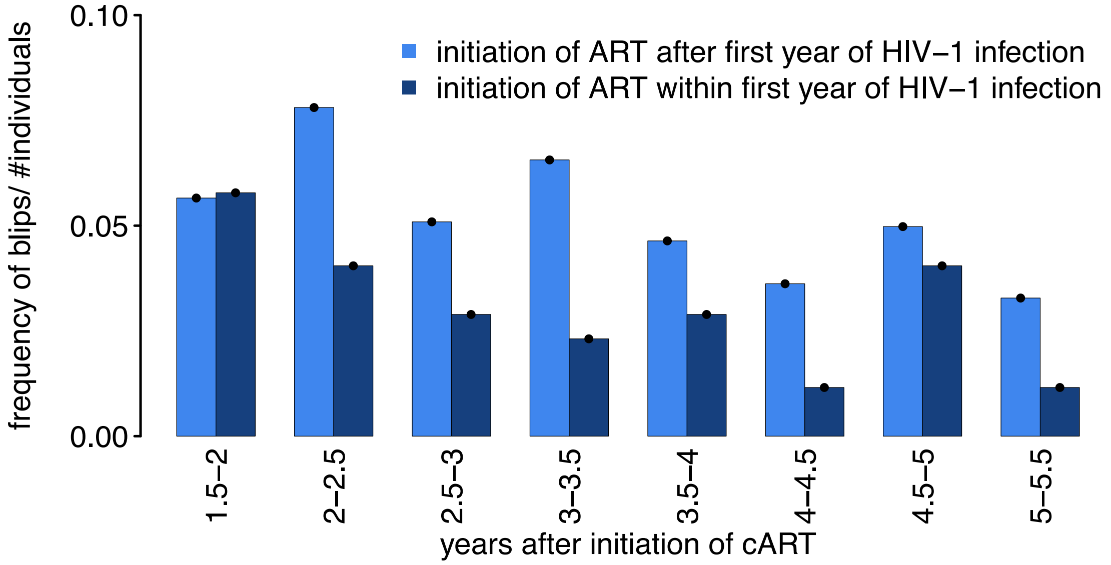

Supplementary Figure 17: *Barplot showing the frequency of blips (more than one per individual possible) divided by the number of individuals and stratified by initiation of ART after the first year of HIV-1 infection (light blue) vs. within the first year of HIV-1 infection (dark blue).*

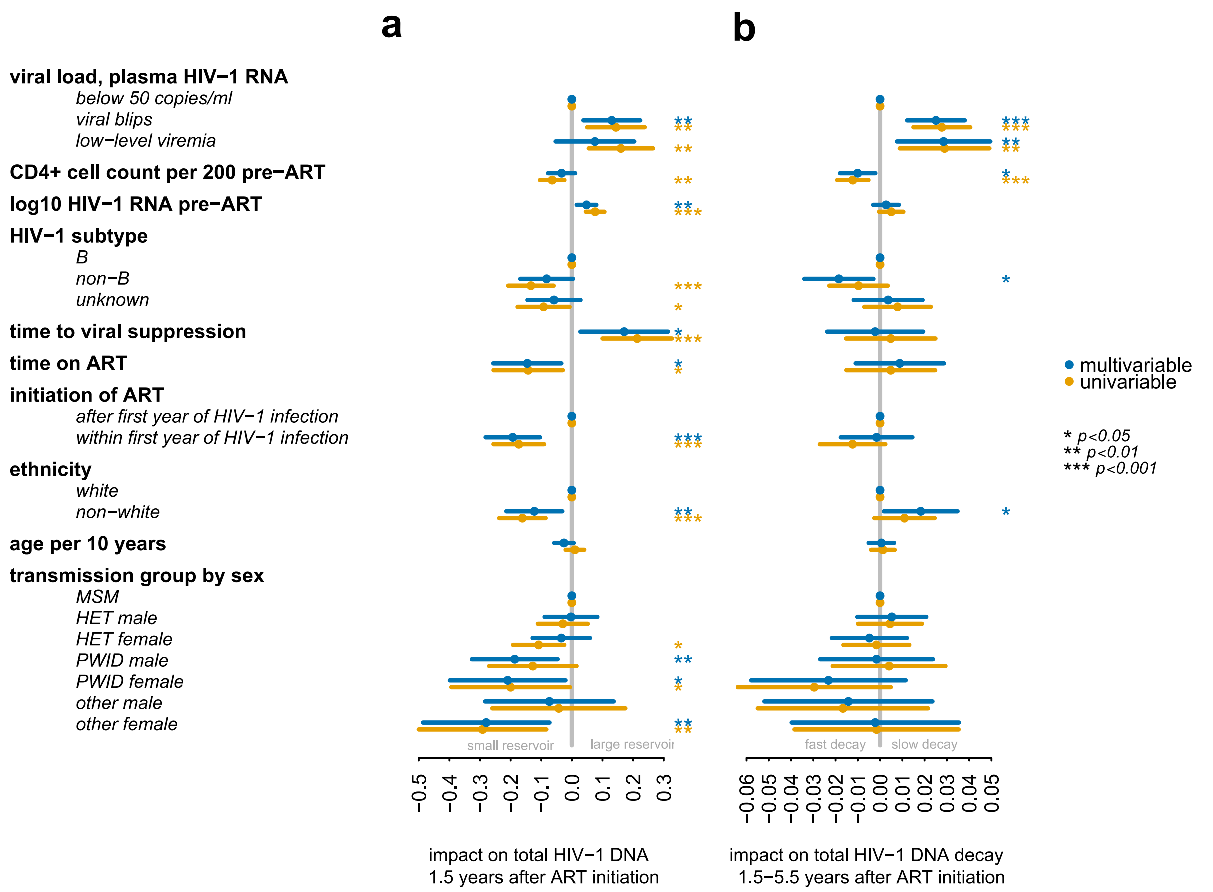

Supplementary Figure 18: Size of viral blips as a determinant of HIV-1 reservoir size and long-term dynamics – missing values were replaced using 10 multiple imputations generated by the R package MICE, which generates multivariate imputations by chained equations. (a) *Coefficient plot showing covariables associated with total HIV-1 DNA levels 1.5 years after initiation of ART and 95% confidence intervals. Viral load <50 HIV-1 RNA copies/ml plasma or low-level viremia refer to the time from 180 days after initiation of ART to the first HIV-1 DNA quantification. Reference was defined as no viral blips, initiation after first year of HIV-1 infection, transmission group MSM, white ethnicity and HIV-1 subtype B.* ***(b)*** *Coefficient plot showing covariables associated with the decay of total HIV-1 DNA levels and 95% confidence intervals. Corrected for initial HIV-1 DNA levels using a spline. Viral load <50 HIV-1 RNA copies/ml plasma or low-level viremia or blips refer to the time between the first and third sample, i.e. 1.5-5.4 years after initiation of ART. Baseline as in panel a. ART, antiretroviral therapy; MSM, men who have sex with men; HET, heterosexual; PWID, people who inject drugs; transmission group other includes unknown and transfusion; time to viral suppression refers to time taken for viral load to drop below 50 HIV-1 RNA copies/ml plasma; CD4+ cell count was measured per 200 cells/µl blood.*

Variable	Coefficient	SE	model	p-value	significance
other female	-0.235695562	0.11400163	Univariate	0.0389623	*
other female	-0.306394498	0.11302401	Multivariate	0.0068348	**
other male	-0.005971679	0.11684355	Univariate	0.95925	
other male	-0.109812849	0.11613628	Multivariate	0.3446242	
PWID female	-0.19132189	0.0971339	Univariate	0.049169	*
PWID female	-0.272750281	0.09843741	Multivariate	0.005704	**
PWID male	-0.123323614	0.07566522	Univariate	0.103466	
PWID male	-0.235370407	0.07224236	Multivariate	0.0011626	**
HET female	-0.101986982	0.04395556	Univariate	0.0205416	*
HET female	-0.026546704	0.04967962	Multivariate	0.5932222	
HET male	-0.007531679	0.04326491	Univariate	0.8618378	
HET male	-0.015655799	0.04556437	Multivariate	0.7312272	
MSM	0	0	Univariate	NA	
MSM	0	0	Multivariate	NA	
age per 10 years	0.013082334	0.01584471	Univariate	0.4092054	
age per 10 years	-0.019479594	0.01687317	Multivariate	0.2486051	
non-white	-0.147616218	0.04016932	Univariate	0.0002514	***
non-white	-0.111577357	0.0483251	Multivariate	0.0211693	*
white	0	0	Univariate	NA	
white	0	0	Multivariate	NA	
within first year of HIV-1 infection	-0.173149486	0.04345832	Univariate	7.29E-05	***
within first year of HIV-1 infection	-0.200341023	0.05170875	Multivariate	0.0001144	***
after first year of HIV-1 infection	0	0	Univariate	NA	
after first year of HIV-1 infection	0	0	Multivariate	NA	
time on ART	-0.125867733	0.05950449	Univariate	0.0346683	*
time on ART	-0.132593651	0.05832038	Multivariate	0.0232222	*
time to viral suppression	0.220646254	0.06043898	Univariate	0.0002758	***
time to viral suppression	0.227930685	0.08311852	Multivariate	0.0062201	**
unknown	-0.065454835	0.04477027	Univariate	0.1440694	
unknown	-0.071907234	0.04786275	Multivariate	0.1333446	
non-B	-0.111804847	0.03929455	Univariate	0.0045328	**
non-B	-0.076739538	0.04550344	Multivariate	0.0920453	
B	0	0	Univariate	NA	
B	0	0	Multivariate	NA	
log10 HIV-1 RNA pre-ART	0.086668044	0.016683	Univariate	2.56E-07	***
log10 HIV-1 RNA pre-ART	0.045175585	0.01718818	Multivariate	0.0087237	**
CD4+ cell count per 200 pre-ART	-0.083831116	0.02183798	Univariate	0.0001321	***
CD4+ cell count per 200 pre-ART	-0.022332284	0.02414566	Multivariate	0.3552599	
low-level viremia	0.208497591	0.05796851	Univariate	0.0003391	***
low-level viremia	0.12260258	0.07157106	Multivariate	0.0870442	
viral blips	0.141110266	0.05049474	Univariate	0.0053036	**

viral blips	0.129934877	0.04793874	Multivariate	0.0068439	**
below 50 copies/ml	0	0	Univariate	NA	
below 50 copies/ml	0	0	Multivariate	NA	

Supplementary Table 1. *Determinants of HIV-1 reservoir size. Table corresponding to the regression analysis on the size of the HIV-1 reservoir 1.5 years after initiation of ART (Figure 3a). Viral load <50 HIV-1 RNA copies/ml plasma or low-level viremia or viral blips refer to the time between the first and third sample, i.e. 1.5-5.4 years after initiation of ART. ART, antiretroviral therapy; MSM, men who have sex with men; HET, heterosexual; PWID, people who inject drugs; transmission group other includes unknown and transfusion; time to viral suppression refers to time taken for viral load to drop below 50 HIV-1 RNA copies/ml plasma; CD4+ cell count was measured per 200 cells/µl blood; SE, standard error.*

Variable	Coefficient	SE	model	p-value	significance
other female	-0.23569556	0.11400163	Univariate	0.0389623	*
other female	-0.3063945	0.11302401	Multivariate	0.0068348	**
other male	-0.00597168	0.11684355	Univariate	0.95925004	
other male	-0.10981285	0.11613628	Multivariate	0.34462424	
PWID female	-0.19132189	0.0971339	Univariate	0.04916897	*
PWID female	-0.27275028	0.09843741	Multivariate	0.005704	**
PWID male	-0.12332361	0.07566522	Univariate	0.10346603	
PWID male	-0.23537041	0.07224236	Multivariate	0.0011626	**
HET female	-0.10198698	0.04395556	Univariate	0.02054156	*
HET female	-0.0265467	0.04967962	Multivariate	0.59322221	
HET male	-0.00753168	0.04326491	Univariate	0.8618378	
HET male	-0.0156558	0.04556437	Multivariate	0.73122718	
MSM	0	0	Univariate	NA	
MSM	0	0	Multivariate	NA	
age per 10 years	0.013082334	0.01584471	Univariate	0.40920544	

age per 10 years	-0.01947959	0.01687317	Multivariate	0.24860513	
non-white	-0.14761622	0.04016932	Univariate	0.00025136	***
non-white	-0.11157736	0.0483251	Multivariate	0.0211693	*
white	0	0	Univariate	NA	
white	0	0	Multivariate	NA	
within first year of HIV-1 infection	-0.17314949	0.04345832	Univariate	7.29E-05	***
within first year of HIV-1 infection	-0.20034102	0.05170875	Multivariate	0.00011442	***
after first year of HIV-1 infection	0	0	Univariate	NA	
after first year of HIV-1 infection	0	0	Multivariate	NA	
time on ART	-0.12586773	0.05950449	Univariate	0.03466829	*
time on ART	-0.13259365	0.05832038	Multivariate	0.02322221	*
time to viral suppression	0.220646254	0.06043898	Univariate	0.00027583	***
time to viral suppression	0.227930685	0.08311852	Multivariate	0.00622006	**
unknown	-0.06545483	0.04477027	Univariate	0.14406941	
unknown	-0.07190723	0.04786275	Multivariate	0.1333446	
non-B	-0.11180485	0.03929455	Univariate	0.00453282	**
non-B	-0.07673954	0.04550344	Multivariate	0.09204526	
B	0	0	Univariate	NA	
B	0	0	Multivariate	NA	
log10 HIV-1 RNA pre-ART	0.086668044	0.016683	Univariate	2.56E-07	***
log10 HIV-1 RNA pre-ART	0.045175585	0.01718818	Multivariate	0.00872367	**
CD4+ cell count per 200 pre-ART	-0.08383112	0.02183798	Univariate	0.00013212	***
CD4+ cell count per 200 pre-ART	-0.02233228	0.02414566	Multivariate	0.35525991	
low-level viremia	0.208497591	0.05796851	Univariate	0.00033913	***
low-level viremia	0.12260258	0.07157106	Multivariate	0.08704416	
viral blips	0.141110266	0.05049474	Univariate	0.00530358	**
viral blips	0.129934877	0.04793874	Multivariate	0.00684388	**
below 50 copies/ml	0	0	Univariate	NA	
below 50 copies/ml	0	0	Multivariate	NA	

Supplementary Table 2. *Determinants of HIV-1 long-term dynamics. Table corresponding to the regression analysis on the decay of the HIV-1 reservoir 1.5 years after initiation of ART (Figure 3b). Corrected for initial HIV-1 DNA levels using a spline. Viral load <50 HIV-1 RNA copies/ml plasma or low-level viremia or viral blips refer to the time between the first and third sample, i.e. 1.5-5.4 years after initiation of ART. ART, antiretroviral therapy; MSM, men who have sex with men; HET, heterosexual; PWID, people who inject drugs; transmission group other includes unknown and transfusion; time to viral suppression refers to time taken for viral load to drop below 50 HIV-1 RNA copies/ml plasma; CD4+ cell count was measured per 200 cells/µl blood; SE, standard error.*
