# Supplementary material for: Determinants of HIV-1 reservoir size and long-term dynamics during suppressive ART
Source: Nat Commun. 2019 Jul 19;10:3193. doi: 10.1038/s41467-019-10884-9 (PMC6642170; doi:10.1038/s41467-019-10884-9)
Supplement: Supplementary file 3 — Reporting Summary [file 41467_2019_10884_MOESM3_ESM.pdf]

nature research

Corresponding author(s): Huldrych F. Günthard

Last updated by author(s): 29.05.2019

Reporting Summary

Nature Research wishes to improve the reproducibility of the work that we publish. This form provides structure for consistency and transparency in reporting. For further information on Nature Research policies, see Authors & Referees and the Editorial Policy Checklist.

Statistics

For all statistical analyses, confirm that the following items are present in the figure legend, table legend, main text, or Methods section.

n/a	Confirmed	
☐	☒	The exact sample size (*n*) for each experimental group/condition, given as a discrete number and unit of measurement
☐	☒	A statement on whether measurements were taken from distinct samples or whether the same sample was measured repeatedly
☐	☒	The statistical test(s) used AND whether they are one- or two-sided<br>*Only common tests should be described solely by name; describe more complex techniques in the Methods section.*
☐	☒	A description of all covariates tested
☐	☒	A description of any assumptions or corrections, such as tests of normality and adjustment for multiple comparisons
☐	☒	A full description of the statistical parameters including central tendency (e.g. means) or other basic estimates (e.g. regression coefficient) AND variation (e.g. standard deviation) or associated estimates of uncertainty (e.g. confidence intervals)
☐	☒	For null hypothesis testing, the test statistic (e.g. *F*, *t*, *r*) with confidence intervals, effect sizes, degrees of freedom and *P* value noted<br>*Give P values as exact values whenever suitable.*
☒	☐	For Bayesian analysis, information on the choice of priors and Markov chain Monte Carlo settings
☒	☐	For hierarchical and complex designs, identification of the appropriate level for tests and full reporting of outcomes
☒	☐	Estimates of effect sizes (e.g. Cohen's *d*, Pearson's *r*), indicating how they were calculated

Our web collection on statistics for biologists contains articles on many of the points above.

Software and code

Policy information about availability of computer code

Data collection	All data was collected on an OpenBIS database.
Data analysis	All data was analyzed using R version 3.5.1. All R code can be made available upon request.

For manuscripts utilizing custom algorithms or software that are central to the research but not yet described in published literature, software must be made available to editors/reviewers. We strongly encourage code deposition in a community repository (e.g. GitHub). See the Nature Research guidelines for submitting code & software for further information.

Data

Policy information about availability of data

All manuscripts must include a data availability statement. This statement should provide the following information, where applicable:

- Accession codes, unique identifiers, or web links for publicly available datasets
- A list of figures that have associated raw data
- A description of any restrictions on data availability

The datasets generated during and/or analyzed during the current study are not publicly available due to privacy reasons, the sensitivities associated with HIV infections, and the representativeness of the dataset. However, a coarse-grained version of the dataset is published with the manuscript as “Supplementary Data 1”.

Field-specific reporting

Please select the one below that is the best fit for your research. If you are not sure, read the appropriate sections before making your selection.

☒ Life sciences ☐ Behavioural & social sciences ☐ Ecological, evolutionary & environmental sciences

For a reference copy of the document with all sections, see nature.com/documents/nr-reporting-summary-flat.pdf

Life sciences study design

All studies must disclose on these points even when the disclosure is negative.

Sample size	Our analyses are based on a longitudinal, observational cohort study (www.shcs.ch). We did not perform sample size calculations, but rather included all SHCS participants fulfilling the inclusion criteria described in the Methods section.
Data exclusions	As recommended for analyses based on observational cohort studies, we followed STrengthening the Reporting of OBservational studies in Epidemiology (STROBE) criteria as shown in figure 1 in detail.
Replication	Replicates of patients' samples were not performed due to the limited number of available cells stored in the SHCS biobank. Multiple controls were included in the droplet digital PCR assays to ensure reproducibility.
Randomization	This study was an observational study and not randomized. However, it relies on data of the Swiss HIV cohort study, which collects a large number of variables. Thus, we were able to correct for many known or suspected confounders.
Blinding	Personnel who measured total HIV-1 DNA were blinded in terms of patients from whom the samples were derived and their respective covariables studied. Investigators were also blinded regarding patient information from the Swiss HIV Cohort Database: patients are annonymized in the SHCS database.

Reporting for specific materials, systems and methods

We require information from authors about some types of materials, experimental systems and methods used in many studies. Here, indicate whether each material, system or method listed is relevant to your study. If you are not sure if a list item applies to your research, read the appropriate section before selecting a response.

Materials & experimental systems

n/a	Involved in the study	
☒	☐	Antibodies
☐	☒	Eukaryotic cell lines
☒	☐	Palaeontology
☒	☐	Animals and other organisms
☐	☒	Human research participants
☐	☒	Clinical data

Methods

n/a	Involved in the study	
☒	☐	ChIP-seq
☒	☐	Flow cytometry
☒	☐	MRI-based neuroimaging

Eukaryotic cell lines

Policy information about cell lines

Cell line source(s)	Sup-T1, ACH-2, and J-Lat (clone 10.6) (NIH AIDS Reagent Program) served as negative or positive controls for the ddPCR assays.
Authentication	Specific authentication was not performed, however, the absence or presence of proviral HIV-1 DNA was confirmed and quantified by qPCR and by HIV-1 integration site analysis for ACH-2 cells.
Mycoplasma contamination	All used cell lines were tested negative for mycoplasma.
Commonly misidentified lines (See ICLAC register)	No commonly misidentified lines were used.

Human research participants

Policy information about studies involving human research participants

Population characteristics	We included 1,057 HIV-infected adults enrolled in the SHCS. Their characteristics are summarized in detail in Table 1 of the manuscript. Systematic selection of these very well characterized and longitudinally studied patients within the SHCS is a key strength of this study.

Recruitment	There was no recruitment for our study. We used biobanked samples and data collected from the pre-existing longitudinal Swiss HIV cohort study as detailed in the methods section. The sole inclusion criteria for participating in the SHCS is to be above the age of 16 and to be HIV-infected. It is an open cohort that continuously recruits individuals from all transmission groups.
Ethics oversight	The SHCS was approved by the ethics committees of the participating institutions (Kantonale Ethikkommission Bern, Ethikkommission des Kantons St. Gallen, Comite Departemental d'Ethique des Specialites Medicales et de Medicine Communataire et de Premier Recours, Kantonale Ethikkommission Zürich, Repubblica et Cantone Ticino–Comitato Ethico Cantonale, Commis- sion Cantonale d'Étique de la Recherche sur l'Être Humain, Ethikkommission beider Basel for the SHCS and Kantonale Ethikkommission Zürich for the ZPHI), and written informed consent was obtained from all participants.

Note that full information on the approval of the study protocol must also be provided in the manuscript.

Clinical data

Policy information about clinical studies

All manuscripts should comply with the ICMJE guidelines for publication of clinical research and a completed CONSORT checklist must be included with all submissions.

Clinical trial registration	not applicable
Study protocol	not applicable
Data collection	not applicable
Outcomes	not applicable
