# Supplementary material for: Determinants of HIV-1 reservoir size and long-term dynamics during suppressive ART
Source: Nat Commun. 2019 Jul 19;10:3193. doi: 10.1038/s41467-019-10884-9 (PMC6642170; doi:10.1038/s41467-019-10884-9)
Supplement: Supplementary file 4 — Description of Additional Supplementary Files [file 41467_2019_10884_MOESM4_ESM.pdf]

Description of Additional Supplementary Files

File Name: Supplementary Data 1
Description: Coarse grained dataset
